# Resilience and Its Association With Activities of Daily Living 3 Months After Stroke

**DOI:** 10.3389/fneur.2022.881621

**Published:** 2022-06-14

**Authors:** Ole Petter Norvang, Anne Eitrem Dahl, Pernille Thingstad, Torunn Askim

**Affiliations:** ^1^Department of Neuromedicine and Movement Science, Faculty of Medicine, NTNU, Norwegian University of Science and Technology, Trondheim, Norway; ^2^Clinical Services, Department of Physiotherapy, St Olavs Hospital, Trondheim University Hospital, Trondheim, Norway; ^3^Stroke Unit, Department of Medicine, St Olavs Hospital, Trondheim University Hospital, Trondheim, Norway

**Keywords:** rehabilitation, recovery, resilience, activities of daily life (ADL), outcome after stroke, prospective observational study, stroke

## Abstract

Independence in basic activities of daily living (ADL) is an important outcome after stroke. Identifying factors associated with independence can contribute to improve post-stroke rehabilitation. Resilience, which is the ability of coping with a serious event, might be such a factor. Still, the impact of resilience and its role in rehabilitation after stroke is poorly investigated. Hence, the purpose of this study was to assess whether resilience assessed early after stroke can be associated with independence in basic ADL 3 months later. Hospitalized patients with a diagnosed acute stroke and a modified Rankin Scale score ≤ 4 were included. Bivariate and multivariate linear regression were applied to assess whether resilience as measured by the Brief Resilience Scale within the first 2 weeks after stroke was associated with basic ADL measured by Barthel Index at 3-month follow-up. Age, sex, fatigue, stroke severity at admission and pre-stroke disability were added as covariates. Sixty-four participants (35 (54.7%) male), aged 75.9 (SD 8.6) years were included 4.3 (SD 2.8) days after stroke. There was no significant change in resilience from baseline 3.1 (SD 0.3) to 3 months later 3.2 (SD 0.5). Resilience was not associated with basic ADL in neither the bivariate (b = 2.01, 95% CI −5.21, 9.23, *p* = 0.580) nor in the multivariate regression models (b = 0.50, 95% CI −4.87, 6.88, *p* = 0.853). Our results showed that resilience remained stable during follow-up. Early measurement of resilience was not associated with independence in basic activities of daily living 3 months after stroke. These results, indicate that resilience is a personal trait not associated with the outcome of physical adversity. However, future research should investigate whether resilience is related to the outcomes of psychosocial adversity after a stroke.

## Introduction

Independence in basic activities of daily living (ADL) which is shown to be associated with improved quality of life for the stroke survivors and reduced burden on the caregivers and the health care system, is an important outcome after stroke (1, 2). Identifying robust and unbiased factors associated with independence in basic ADL is, therefore, important in stroke rehabilitation.

Independence in ADL involves a complex construct consisting of physical, psychological and social factors. Coping skills, level of active social participation and number of social ties may all be important in dealing with the stroke and has been shown to improve both well-being and functioning 3 months after discharge (3, 4).

Resilience, which is a process of “bouncing back” or coping with a serious event is defined as “an outcome of successful adaptation to adversity, revealed by either sustainability, recovery, or both” (5, 6). It involves adapting personal coping strategies and the use of social network and has increasingly been recognized as a protective factor for health (6). For instance, resilience has shown to have a moderating and mediating role in the associations between coping style and uncertainty in illness (7), and to protect against unfavorable cardiometabolic outcomes that are otherwise more likely in stroke patients (8). A higher level of resilience has also been associated with better cardiovascular outcomes (9). However, it is not clear whether resilience should be considered as a personal trait or a learnt behavior (5).

The body of literature within this field, covering both human and animal studies, has increased substantially over the past decade giving a better understanding of the neurophysiological and neuropsychological mechanisms of resilience (10). The neurophysiological mechanisms involves the medial prefrontal cortex, the hippocampus, and the ventral tegmental area (VTA), all involved in coping with stress. A dysfunction in these areas have been shown to affect resilience (10–12). Resilience also includes a deeper understanding of the relationship between intrapersonal, interpersonal and socio-ecological constructs has therefore been highlighted as important to understanding and fostering of resilience in stroke survivors (13).

Following a stroke, less adaptive psychological factors has shown to be negatively associated with participation over time (14), while resilience has shown to act as an independent predictor of quality of life and physical independence (15–17). Therefore, resilience has been highlighted as a factor to consider in optimizing rehabilitation early after a stroke (7, 18–20). In the study by Gyawali et al. (14), resilience was associated with stroke outcomes in the chronic phase. However, the relationship between resilience and associated variables is complex and, to a large degree, unexplored (20, 21). Understanding the role of resilience early after a stroke may therefore provide insight into both short- and long-term recovery and in regaining ADL function.

The aim of this study was therefore to assess whether resilience assessed within the first 2 weeks after a stroke can be associated with independence in basic ADL 3 months later.

We hypothesized that a higher score on the Brief Resilience scale would be associated with independence in basic ADL 3 months after a stroke.

## Materials and Methods

### Study Design

This study had a prospective longitudinal cohort design, with inclusion and baseline assessment within the first 2 weeks after stroke onset and a follow-up assessment 3 months later (±2 weeks).

### Study Settings and Procedure

Participants were treated in an evidence-based comprehensive stroke unit, with a special focus on independence in daily life after a stroke (22). The stroke unit has a multidisciplinary approach focusing on early mobilization, physiological homeostasis, nutrition and fluid intake to provide best possible treatment (23, 24). While hospitalized, participants were screened for inclusion into the study by an experienced physiotherapist. Recruitment took place 3 days a week. Only patients available for inclusion on one of these days were invited to participate.

All baseline assessments were performed during hospital stay within 1 day from inclusion in the study. This included the baseline assessments, the independent variable (the Brief Resilience Scale), and all covariates included in the regression analysis. Participants were invited back to the hospital for the follow-up assessment at the outpatient clinic 3 months later. All assessments were performed by the same physiotherapist at both time points.

In line with the Norwegian guidelines for treatment and rehabilitation after stroke (22), participants were discharged from the stroke unit in accordance with their physical and cognitive level for further follow-up in the primary health care system to either a rehabilitation center, outpatient clinic or at home with or without organized home-based treatment.

### Study Sample

Patients admitted to the stroke unit at St Olavs Hospital, Trondheim University Hospital in Norway were asked for their participation. Patients with first-ever or recurrent acute ischemic or hemorrhagic stroke were eligible for inclusion if the onset of stroke was within 14 days post-stroke, and their modified Rankin Scale (mRS) score (25) was 0–4 points. Aphasia or language problems were accepted for inclusion as long as the patients scored 4–6 points on the item “orientation” on the Scandinavian Stroke Scale (SSS) (26), and they were capable of providing informed consent. However, persons with a life expectancy of <6 months, such as advanced and progressive cancer and unstable heart conditions, or serious impairments that could have significant impact on a functional outcome or unstable medical condition after an acute stroke were excluded.

### Baseline Assessments

At baseline, age and sex, days from stroke to inclusion and the degree of disability prior to the stroke (measure by the mRS) were scored. mRS is ranging from 0 (no symptoms) to 5 (severe disability), while 6 denotes death. The scale has shown strong validity and reliability (27). From the medical records of the participants the type and location of stroke, severity of the stroke measured by the SSS, and possible reperfusion treatment were assessed. The SSS, ranging from 0 (worst) to 58 (best) has shown to be a valid measure of stroke severity (28, 29).

The baseline assessments also included the degree of disability, measured by the mRS and the Barthel Index (BI) and fatigue measured by the Fatigue Severity Scale (FSS-7). Fatigue is reported to be up to three times higher three months after a stroke than in the general population (30). FSS-7 has shown to be reliable and valid in the Norwegian stroke population (31, 32). Each item was scored on a 7-point Likert scale, with a total FSS score ranging from 7 (best) to 49 (worst). The total score was averaged to yield a score from 1.0 to 7.0. Higher scores indicate higher fatigue levels. FSS-7 was completed by self-report.

### Dependent Variable

The Barthel Index obtained at 3-month follow-up was added as the dependent variable to assess independence in basic ADL, ranging from 0 (worst) to 100 (best) (33). Barthel Index has proven good reliability in the stroke population (34), however the documented ceiling effect is a weakness (35).

### Independent Variables

The Brief Resilience Scale (BRS) obtained at baseline was used to measure the ability to cope with a serious event. It consists of six items with five different response categories, giving a total score ranging from 6–30. The total score is further divided by the number of items answered giving an average score of 1 (worst) to 5 (best). BRS has shown to be a reliable tool to assess an individuals' ability to recover from stressful circumstances (36, 37). The BRS used in its this project was the original version in English. The BRS questionnaire was completed in a collaboration between the participant and the physiotherapist.

### Covariates

Covariates included in the regression analysis were identified and selected from previous literature. These were age, sex, fatigue at baseline, degree of independence prior to stroke, and stroke severity at admission (38–40).

### Statistical Analysis

All data were analyzed using IBM SPSS Statistics version 25. Unless otherwise stated, demographic data were reported as mean values and standard deviation (SD) for all participants, retrieved from the participants‘ medical record. Residuals were visually inspected for normal distribution by Q-Q plots with both dependent and independent variables showing a normal distribution. Homoscedasticity was controlled through a scatter plot of the residuals. Paired sample *t*-tests were applied to study changes in ADL and resilience from baseline to 3 months later. Linear regression was used to analyze associations with Barthel Index at 3 months as the dependent variable and resilience at baseline as independent variables in both bivariate and multivariate regression analysis. Age, sex, fatigue at baseline, stroke severity at admission and the modified Rankin Scale prior to inclusion were added as covariates. The covariates were selected based on clinical judgement and the literature. We checked for collinearity with VIF <10 and correlation values below 0.9. Significance level was set at *p* < 0.05 for all associations.

## Results

**Figure 1:**
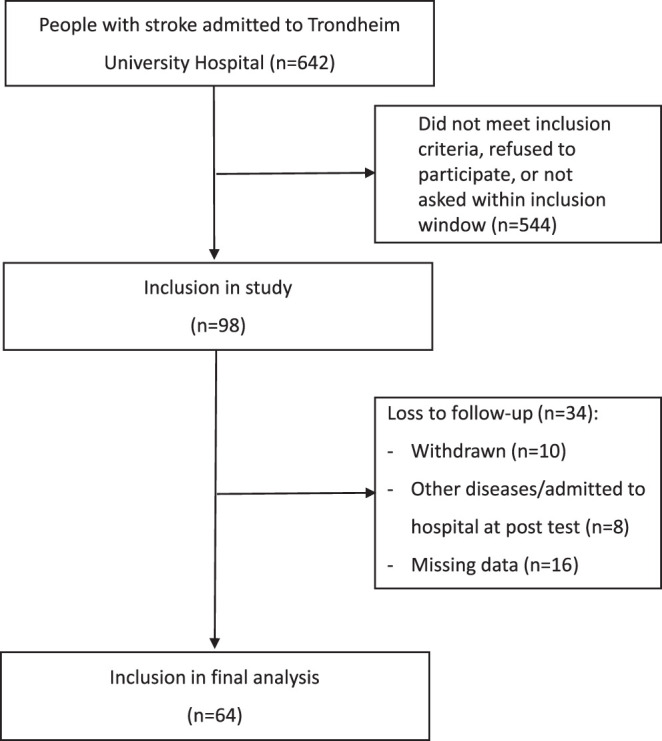
Flow chart of the participants.

As displayed in Figure 1, a total of 98 people accepting participation were included, representing about 15% of those admitted to the stroke unit during the recruitment period. Out of these, 34 participants were lost to follow-up, mainly due to missing data (*n* = 15) and participants who withdraw from the study (*n* = 10).

There were no statistically significant differences in baseline characteristics between those included in the analysis (*n* = 64) and those being lost-to-follow up (*n* = 34).

Table 1 shows the baseline characteristics of the sixty-four participants, 35 (54.7%) males, with a mean age of 75.9 (SD = 8.6) years included in the analyses. Of these, 14 (21.9%) persons underwent reperfusion treatment. Participants were hospitalized for an average of 6.7 (SD = 3.4) days and included in the study 4.3 (SD = 2.8) days after onset of symptom. Mean score of the Scandinavian Stroke Scale was 45.8 (SD = 8.6) at admission, indicating mild-to-moderate strokes. Mean degree of disability at baseline was 2.8 (SD = 0.9) points on the modified Rankin Scale. Participants were discharged, in accordance with their physical and cognitive level, to a rehabilitation center, *n* = 21 (32.8%), an outpatient clinic, *n* = 13 (20.3%), or home-based treatment, *n* = 30) (46.9%).

**Table 1:** Baseline characteristics (*n* = 64).

		**Range**
Age (years), mean (SD)	75.9 (8.7)	60–99
Days hospitalized, mean (SD)	6.7 (3.4)	2–16
Days from stroke to inclusion, mean (SD)	4.3 (2.8)	1–12
Scandinavian Stroke Scale (0–58) at admission, mean (SD)	45.8 (8.6)	6–58
Barthel Index, mean (SD)	84.9 (14.6)	40–100
Male sex, *n* (%)	35 (54.7)	
Reperfusion treatment, *n* (%)	14 (21.9)	
**Types of stroke**, ***n*** **(%)**		
Ischemic stroke	50 (78.1)	
Hemorrhagic stroke	4 (6.3)	
Unclassified stroke	10 (15.6)	
Modified Rankin Scale (mRS) (0–6), mean (SD)	2.8 (0.9)	
mRS 0, *n* (%)	0 (0.0)	
mRS 1, *n* (%)	3 (4.7)	
mRS 2, *n* (%)	26 (40.6)	
mRS 3, *n* (%)	17 (26.6)	
mRS 4, *n* (%)	18 (28.1)	
**Side affected by the stroke**, ***n*** **(%)**		
Left	30 (46.9)	
Right	24 (37.5)	
Bilateral	4 (6.3)	
Unclear	6 (9.3)	
**Discharge destination**, ***n*** **(%)**		
Rehabilitation center	21 (32.8)	
Outpatient clinic	13 (20.3)	
Home-based treatment	30 (46.9)	

*mRS 0, no symptoms; mRS 1, no significant disability; mRS 2, slight disability; but needs no assistance; mRS 3, moderate disability, require some help but walk without assistance; mRS 4, moderately severe disability; unable to walk without assistance; mRS 5; severe disability, requires constant nursing care; mRS 6, dead*.

Mean Barthel Index score at baseline was 74.5 (SD = 12.9) points for those discharged to inpatient rehabilitation, 93.9 (SD = 6.8) points for those discharged to outpatient rehabilitation, and 88.3 (SD = 14.3) points for those discharged to homebased rehabilitation, respectively.

**Figure 2:**
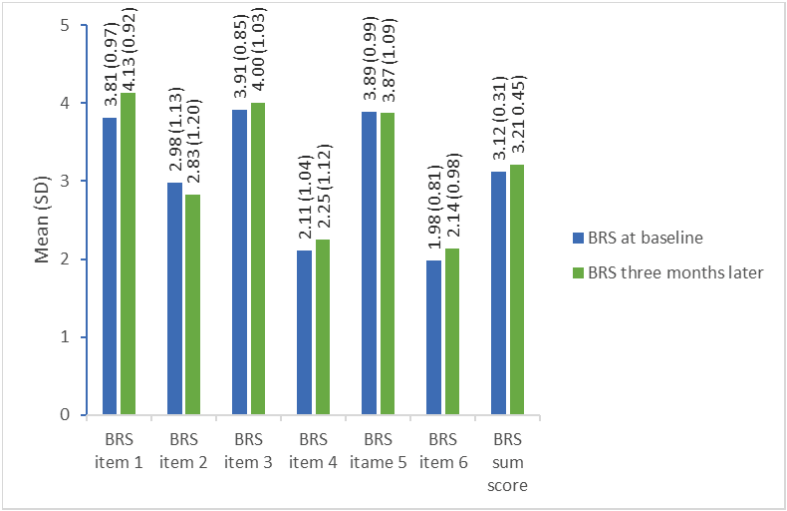
Single item and full-score resilience measured by the Brief Resilience Scale at baseline and 3-month follow-up (*n* = 64). BRS item 1: I tend to bounce back quickly after hard times (1 = strongly disagree to 5 = strongly agree), BRS item 2: I have a hard time making it through stressful events (1 = strongly agree to 5 = strongly disagree), BRS time 3: It does not take me long to recover from a stressful event (1 = strongly disagree to 5 = strongly agree), BRS item 4: It is hard for me to snap back when something bad happens (1 = strongly agree to 5=strongly disagree), BRS item 5: I usually come through difficult times with little trouble (1 = strongly disagree to 5 = strongly agree), BRS time 6: I tend to take a long time to get over set-backs in my life (1 = strongly agree to 5 = strongly disagree).

The mean degree of independence in basic ADL improved significantly (*p* < 0.001) from 84.9 (SD = 14.6) points on the Barthel Index during hospitalization to 95.0 (SD = 9.0) 3 months after the stroke. There was no significant change in overall resilience from baseline to 3 months later (mean = 3.1 (SD = 0.3) versus mean = 3.2 (SD = 0.5) points). This was also the trend for each of the items in the BRS with only minor changes from baseline to follow-up, despite item 1 (I tend to bunch back quickly after hard times) which increased from 3.81 (SD = 0.97) at baseline to 4.13 (SD = 0.32) at 3-month follow-up. The mean scores for each item and the overall score at baseline and follow-up are displayed in Figure 2.

**Figure 3:**
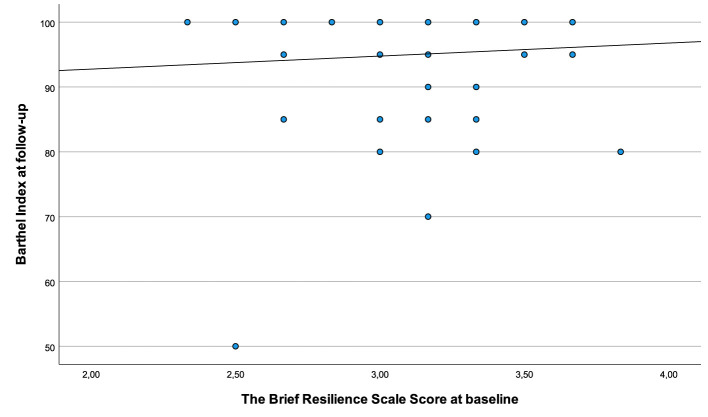
Associations between resilience scored during hospitalization and independence in basic ADL 3 months post-stroke.

Figure 3 shows the association between resilience at baseline and independence in basic ADL, as measured by Barthel Index at 3 months later. The plots reveal a ceiling effect of the Barthel Index, with the majority of the participants scoring higher than 90 points.

Table 2 shows the results from the regression analysis. Resilience measured at baseline did not show a statically significant association with independence in basic ADL after 3 months in neither the bivariate (b = 2.01, 95% CI −5.21, 9.23, *p* = 0.580) nor in the multivariate analysis (b = 0.50, 95% CI −4.87, 6.88, *p* = 0.853), with an adjusted *R*^2^ of 0.461. Age and mRS score prior to the stroke were the covariates with the strongest association with Barthel Index 3 months after stroke.

**Table 2:** Bivariate and multivariate linear regression analyses for association between resilience and independence in basic ADL after stroke (*n* = 64).

**Independence in basic ADL measured by Barthel Index 3 months after stroke**						
	**Bivariate analyses**			**Multivariate analyses**		
	**b**	**95% CI**	***p***	**b**	**95% CI**	***p***
Resilience	2.01	−5.21 to 9.23	0.580	0.50	−4.87 to 6.88	0.853
**Covariates**						
FSS-7 at baseline	0.01	−0.19 to 0.19	0.986	0.10	−0.06 to 0.26	0.219
Age	−0.68	−0.88 to −0.48	<0.001	−0.50	−0.73 to −0.28	<0.001
Female sex	−4.41	−8.80 to −0.02	0.049	−2.27	−6.22 to 0.97	0.149
mRS prior to stroke	−7.00	−9.20 to −4.08	<0.001	−4.09	−7.23 to −0.95	0.012
SSS at admission	0.13	−0.14 to 0.39	0.346	−0.02	−0.22 to 0.18	0.835

*ADL, Activities of Daily Living; FSS-7, Fatigue Severity Scale 7-item; mRS, Modified Rankin Scale; SSS, Scandinavian Stroke Scale*.

## Discussion

In contrast to our hypothesis, the results from this study showed that resilience score obtained within 2 weeks after stroke was not associated with independence in basic ADL 3 months later.

Generally, resilience could be described as a good outcome despite adversity and risk (41). Our results showed no significant association between resilience at baseline and good outcome in basic ADL 3 months later. This result could indicate that the impact of resilience might not apply to physical adversity, like basic ADL, but is related to psychosocial adversity as shown by several other researchers (6, 14, 18).

Another possible explanation for the lack of association could be the study sample with a relatively high Barthel Index score at 3-month follow-up. The scatter plot in Figure 3, is also confirming a ceiling effect and a low spread of the Barthel Index score in relation to resilience. Furthermore, the baseline characteristics showing mild-to-moderate stroke severity at inclusion suggest a relatively well-functioning selected group. It is, therefore, possible that including a more heterogenous sample with more severely affected stroke survivors also would have given a different result.

The significant improvement in Barthel Index from baseline to 3-month follow-up is in line with previous research showing that the first 3 months after a stroke is when spontaneous and behavioral recovery is at its highest (42, 43). In contrast, the Brief Resilience Scale showed no change during this period, supporting the assumption that resilience is a personal trait (44), which might be independent of the recovery process. The mean resilience score of 3.1 (SD 0.3) at baseline is indicating an average neutral response to the statements in BRS in this population. It would have been interesting to know their resilience score prior to the stroke. However, as indicated by Figure 2 the scores were close to 4 points for the positively worded items (item 1, 3, and 5) and below three points for the negatively worded items (item 2, 4, and 6), which may indicate that the participants were not able to fully understand the wording and scoring of all items. The Brief Resilience Scale does not have a specified change in score that would be clinically meaningful but has previously shown an excellent construct validity and an excellent internal consistency with alphas >0.70 and <0.95 (37). Even though the BRS has been validated in several populations with chronic diseases (36, 37) our study indicates that it needs to be validated also in the stroke population.

### Methodological Considerations

This is the first study to assess the association between resilience in the early phase and basic ADL 3 months later in a stroke population. Assessing resilience within 2 weeks after a stroke is assumed to give highly valid response to the ability to bounce back from a serious event, which should be considered a major strength of this study. Another strength was that all included participants underwent treatment in an evidence-based stroke unit with a focus on early rehabilitation to regain independence in basic ADL. The multidisciplinary approach with focus on physiological homeostasis, nutrition, and fluid intake and also on early mobilization and goal achievement (23, 24), might have facilitated positive coping strategies in a stressful situation (45).

Ninety-eight subjects accepting participation represented about 15% of patients admitted to the stroke unit during the recruitment period. The low recruitment rate was caused by patients not meeting our inclusion criteria, failing to be screened inside the inclusion window, or refused participation. Another important reason for the low inclusion rate was because the physiotherapist responsible for inclusion was working part-time, which might have increased the risk of failing to include patients with a short hospital stay. Unfortunately, we do not have a detailed screening log reporting the specific reason for being excluded from the study. The low recruitment rate might represent a selection bias. However, comparison with data from the Norwegian stroke registry suggests that our participants were comparable to most stroke survivors in Norway regarding stroke severity and degree of disability (46). Of the 98 participants first included in the study, 34 (34%) were lost to follow-up. However, secondary analysis of baseline characteristics of age, sex, modified Rankin Scale prior to the stroke, stroke severity, functional impairment, and independence in basic ADL did not show any clinical or statistical difference between those included and excluded from the study. Therefore, we would argue that the participants included in the final analysis were representative of those originally included in the study.

We applied rather broad inclusion criteria to ensure inclusion of a population representing the heterogeneity of stroke survivors. However, in line with several other stroke studies (47, 48) the baseline characteristics (Table 1) suggest that participants had only mild-to-moderate disability after stroke, and with the ceiling effect of the Barthel Index after 3 months further underlines this (Figure 3). The ceiling effect is likely to influence on our results with a low variance making associations less likely. This should be considered when interpreting our results. Including stroke survivors with more severe strokes and without the ability to move independently could have given different results. Even though, the Barthel Index has been widely used as an assessment tool for ADL function for stroke survivors, adding a more extensive and responsive measure like the Functional Independent Measure (49) or the Stroke Impact Scale (50) would be more appropriate to applied in a future study.

The inclusion criteria allowed both first time- and recurrent strokes to be included in the study. Participants with a recurrent stroke may have suffered from impairments such as movement disorders or fatigue prior to testing, which could influence on the results. This variation has been accounted for by adjusting for pre-stroke function as measured by the modified Rankin Scale in the multivariate regression analysis. However, in future studies with a larger sample size, a sub-group analysis differentiating between first ever and recurrent strokes could add additional information about sub-groups of stroke patients.

The relatively wide inclusion window ranging from 1 to 12 days after stroke onset may influence on the results, as spontaneous recovery is at its highest the first days and weeks after a stroke. This inclusion window was chosen to recruit as many participants as possible. However, the average day from stroke onset to inclusion was 4.3 (SD 2.8) days, indicating that most participants were included within the first week after the stroke.

The linear regression was chosen instead of dichotomizing Barthel Index in order to maintain statistical power given the relatively small sample size. We chose to add 5 different covariates to the analysis. These were chosen based on the literature and clinical judgment. Sex has previously been questioned as a predictor of ADL outcome after a stroke (51). However, the bivariate analysis showed a borderline significance level (*p* = 0.049), which was an argument to include sex as a covariate in the multivariate analysis.

In conclusion, our results showed that early measurement of resilience was not associated with independence in basic activities of daily living 3 months after stroke, indicating that resilience is a personal trait which do not have an impact on the outcome of physical adversity. However, future research should investigate whether resilience is related to the outcomes of psychosocial adversity after stroke.

## Data Availability Statement

Due to Norwegian regulations and conditions for informed consent, the dataset will not be publicly available before it can be anonymized in 2025.

## Ethics Statement

The studies involving human participants were reviewed and approved by Central Norway Regional Committee for Medical and Health Research Ethics (REC number 2011/2517). The patients/participants provided their written informed consent to participate in this study.

## Author Contributions

TA and AED planned the study. AED recruited and tested patients. AED, OPN, and TA planned and processed data. OPN, PT, and TA performed the statistics by analyzing the data and interpreted the results. The manuscript was written by OPN and TA. All authors critically reviewed and approved the manuscript before it was submitted.

## Funding

This study was funded by the Norwegian University of Science and Technology.

## Conflict of Interest

The authors declare that the research was conducted in the absence of any commercial or financial relationships that could be construed as a potential conflict of interest.

## Publisher's Note

All claims expressed in this article are solely those of the authors and do not necessarily represent those of their affiliated organizations, or those of the publisher, the editors and the reviewers. Any product that may be evaluated in this article, or claim that may be made by its manufacturer, is not guaranteed or endorsed by the publisher.

## References

[1] Legg LA Lewis SR Schofield-Robinson OJ Drummond A Langhorne P. Occupational therapy for adults with problems in activities of daily living after stroke. Cochrane Database Syst Rev. (2017) 7:Cd003585. 10.1002/14651858.CD003585.pub3 28721691 PMC6483548

[2] Bohannon R Andrews A Smith M. Rehabilitation goals of patients with hemiplegia. Int J Rehabil Res. (1988) 11:181–4. 10.1097/00004356-198806000-00012

[3] Berges IM Seale GS Ostir GV. The role of positive affect on social participation following stroke. Disabil Rehabil. (2012) 34:2119–23. 10.3109/09638288.2012.673684 22506691

[4] Kamat R Depp CA Jeste DV. Successful aging in community seniors and stroke survivors: current and future strategies. Neurol Res. (2017) 39:566–72. 10.1080/01616412.2017.1322348 28468534

[5] Zautra AJ Hall JS Murray KE. The resilience solutions G. Resilience: a new integrative approach to health and mental health research. Health Psychol Rev. (2008) 2:41–64. 10.1080/17437190802298568

[6] Hildon Z Montgomery SM Blane D Wiggins RD Netuveli G. Examining resilience of quality of life in the face of health-related and psychosocial adversity at older ages: what is “right” about the way we age? Gerontologist. (2010) 50:36–47. 10.1093/geront/gnp067 19549715

[7] Han ZT Zhang HM Wang YM Zhu SS Wang DY. Uncertainty in illness and coping styles: moderating and mediating effects of resilience in stroke patients. World J Clin Cases. (2021) 9:8999–9010. 10.12998/wjcc.v9.i30.8999 34786383 PMC8567502

[8] Nishimi KM Koenen KC Coull BA Chen R Kubzansky LD. Psychological resilience predicting cardiometabolic conditions in adulthood in the Midlife in the United States Study. Proc Natl Acad Sci USA. (2021) 118:e2102619118. 10.1073/pnas.2102619118 34341103 PMC8364125

[9] Park JW Mealy R Saldanha IJ Loucks EB Needham BL Sims M. Multilevel resilience resources and cardiovascular disease in the United States: a systematic review and meta-analysis. Health Psychol. (2021) 41:278–90. 10.1037/hea0001069 34138614 PMC8678382

[10] Liu H Zhang C Ji Y Yang L. Biological and psychological perspectives of resilience: is it possible to improve stress resistance? Front Hum Neurosci. (2018) 12:326. 10.3389/fnhum.2018.00326 30186127 PMC6110926

[11] Franklin TB Saab BJ Mansuy IM. Neural mechanisms of stress resilience and vulnerability. Neuron. (2012) 75:747–61. 10.1016/j.neuron.2012.08.016 22958817

[12] Russo SJ Murrough JW Han MH Charney DS Nestler EJ. Neurobiology of resilience. Nat Neurosci. (2012) 15:1475–84. 10.1038/nn.3234 23064380 PMC3580862

[13] Anderson MA Buffo C Ketcher D Nguyen H MacKenzie JJ Reblin M. Applying the rise model of resilience in partners post-stroke: a qualitative analysis. Ann Behav Med. (2022) 56:270–81. 10.1093/abm/kaab053 34228090 PMC8887576

[14] Gyawali P Chow WZ Hinwood M Kluge M English C Ong LK. Opposing associations of stress and resilience with functional outcomes in stroke survivors in the chronic phase of stroke: a cross-sectional study. Front Neurol. (2020) 11:230. 10.3389/fneur.2020.00230 32390923 PMC7188983

[15] Lo Buono V Corallo F Bramanti P Marino S. Coping strategies and health-related quality of life after stroke. J Health Psychol. (2017) 32:16–28. 10.1177/1359105315595117 26220458

[16] Liu Z Zhou X Zhang W Zhou L. Factors associated with quality of life early after ischemic stroke: the role of resilience. Top Stroke Rehabil. (2019) 26:335–41. 10.1080/10749357.2019.1600285 30957683

[17] Liu Z Zhou X Zhang W Zhou L. Resilience is an independent correlate of the course of quality of life in patients with first-ever ischemic stroke. Int Psychogeriatr. (2021) 33:567–75. 10.1017/S1041610220000587 32418551

[18] van Rijsbergen MWA Mark RE Kop WJ de Kort PLM Sitskoorn MM. Psychological factors and subjective cognitive complaints after stroke: beyond depression and anxiety. Neuropsychol Rehabil. (2018) 29:1–14. 10.1080/09602011.2018.1441720 29502474

[19] Cheong MJ Kang Y Kang HW. Psychosocial factors related to stroke patients' rehabilitation motivation: a scoping review and meta-analysis focused on South Korea. Healthcare. (2021) 9:1211. 10.3390/healthcare9091211 34574985 PMC8471222

[20] Love MF Wood GL Wardell DW Beauchamp JES. Resilience and associated psychological, social/cultural, behavioural, and biological factors in patients with cardiovascular disease: a systematic review. Eur J Cardiovasc Nurs. (2021) 20:604–17. 10.1093/eurjcn/zvaa008 34223625

[21] Fuller-Thomson E Jensen LA. Flourishing after a stroke: a nationally representative portrait of resilience and mental health among older Canadians. J Aging Health. (2020) 32:308–16. 10.1177/0898264318822228 30624141

[22] Norwegian Directorate of Health. Nasjonal faglig retningslinje for behandling og rehabilitering ved hjerneslag. Oslo, Norway: Norwegian Directorate of Health (2017) (in Norwegian).

[23] Indredavik B Bakke F Slordahl SA Rokseth R Haheim LL. Treatment in a combined acute and rehabilitation stroke unit: which aspects are most important? Stroke. (1999) 30:917–23. 10.1161/01.STR.30.5.917 10229720

[24] Langhorne P Pollock A. What are the components of effective stroke unit care? Age Ageing. (2002) 31:365–71. 10.1093/ageing/31.5.365 12242199

[25] Wilson JT Hareendran A Hendry A Potter J Bone I Muir KW. Reliability of the modified Rankin Scale across multiple raters: benefits of a structured interview. Stroke. (2005) 36:777–81. 10.1161/01.STR.0000157596.13234.95 15718510

[26] Stubbs PW Mortensen J. Clinimetrics: the scandinavian stroke scale. J Physiother. (2020) 66:132. 10.1016/j.jphys.2019.08.010 31521548

[27] Banks JL Marotta CA. Outcomes validity and reliability of the modified Rankin scale: implications for stroke clinical trials: a literature review and synthesis. Stroke. (2007) 38:1091–106. 10.1161/01.STR.0000258355.23810.c6 17272767

[28] Ghandehari K. Challenging comparison of stroke scales. J Res Med Sci. (2013) 18:906d10. 24497865 PMC3897078

[29] Luvizutto GJ Monteiro TA Braga G Pontes-Neto OM de Lima Resende LA Bazan R. Validation of the scandinavian stroke scale in a multicultural population in Brazil. Cerebrovasc Dis Extra. (2012) 2:121–6. 10.1159/000345948 23341824 PMC3551386

[30] Egerton T Hokstad A Askim T Bernhardt J Indredavik B. Prevalence of fatigue in patients 3 months after stroke and association with early motor activity: a prospective study comparing stroke patients with a matched general population cohort. BMC Neurol. (2015) 15:181. 10.1186/s12883-015-0438-6 26444541 PMC4596493

[31] Johansson S Kottorp A Lee KA Gay CL Lerdal A. Can the Fatigue Severity Scale 7-item version be used across different patient populations as a generic fatigue measure–a comparative study using a Rasch model approach. Health Qual Life Outcomes. (2014) 12:24. 10.1186/1477-7525-12-24 24559076 PMC3936846

[32] Nadarajah M Mazlan M Abdul-Latif L Goh HT. Test-retest reliability, internal consistency and concurrent validity of fatigue severity scale in measuring post-stroke fatigue. Eur J Phys Rehabil Med. (2017) 53:703–9. 10.23736/S1973-9087.16.04388-4 27768012

[33] Mahoney FI Barthel DW. Functional evaluation. The barthel index. Md State Med J. (1965) 14:61e Med Jluati1037/t02366-000 14258950

[34] Green J Forster A Young J, A. test-retest reliability study of the barthel index, the rivermead mobility index, the nottingham extended activities of daily living scale and the frenchay activities index in stroke patients. Dis Rehabil. (2001) 23:670–6. 10.1080/09638280110045382 11720117

[35] Barak S Duncan PW. Issues in selecting outcome measures to assess functional recovery after stroke. NeuroRx. (2006) 3:505–24. 10.1016/j.nurx.2006.07.009 17012065 PMC3593403

[36] Smith BW Dalen J Wiggins K Tooley E Christopher P Bernard J. The brief resilience scale: assessing the ability to bounce back. Int J Behav Med. (2008) 15:194–200. 10.1080/10705500802222972 18696313

[37] Windle G Bennett KM Noyes J, A. methodological review of resilience measurement scales. Health Qual Life Outcomes. (2011) 9:8. 10.1186/1477-7525-9-8 21294858 PMC3042897

[38] Cumming TB Packer M Kramer SF English C. The prevalence of fatigue after stroke: a systematic review and meta-analysis. Int J Stroke. (2016) 11:968–77. 10.1177/1747493016669861 27703065

[39] Reeves MJ Bushnell CD Howard G Gargano JW Duncan PW Lynch G. Sex differences in stroke: epidemiology, clinical presentation, medical care, and outcomes. Lancet Neurol. (2008) 7:915–26. 10.1016/S1474-4422(08)70193-5 18722812 PMC2665267

[40] Shavelle RM Brooks JC Strauss DJ Turner-Stokes L. Life expectancy after stroke based on age, sex, and rankin grade of disability: a synthesis. J Stroke Cerebrovasc Dis. (2019) 28:104450. 10.1016/j.jstrokecerebrovasdis.2019.104450 31676160

[41] Masten AS. Ordinary magic. Resilience processes in development. Am Psychol. (2001) 56:227–38. 10.1037/0003-066X.56.3.227 11315249

[42] Jorgensen HS Nakayama H Raaschou HO Vive-Larsen J Stoier M Olsen TS. Outcome and time course of recovery in stroke. Part II: time course of recovery the copenhagen stroke study. Arch Phys Med Rehabil. (1995) 76:406–12. 10.1016/S0003-9993(95)80568-0 7741609

[43] Bernhardt J Hayward KS Kwakkel G Ward NS Wolf SL Borschmann K. Agreed definitions and a shared vision for new standards in stroke recovery research: the Stroke Recovery and Rehabilitation Roundtable taskforce. Int J Stroke. (2017) 12:444–50. 10.1177/1747493017711816 28697708

[44] Joyce S Shand F Tighe J Laurent SJ Bryant RA Harvey SB. Road to resilience: a systematic review and meta-analysis of resilience training programmes and interventions. BMJ Open. (2018) 8:e017858. 10.1136/bmjopen-2017-017858 29903782 PMC6009510

[45] Suddick KM Cross V Vuoskoski P Stew G Galvin KT. Holding space and transitional space: stroke survivors' lived experience of being on an acute stroke unit. A hermeneutic phenomenological study. Scand J Caring Sci. (2021) 35:104–14. 10.1111/scs.12824 32065418 PMC7984029

[46] Fjærtoft H Indredavik B Mørch B Phan A Skogseth-Stephani R Halle KK. Årsrapport 2017. Trondheim: Norsk Hjerneslagregister (2017) (in Norwegian).

[47] Avert Trial Collaboration group. Efficacy and safety of very early mobilisation within 24 h of stroke onset (AVERT): a randomised controlled trial. Lancet. (2015) 386:46–55. 10.1016/S0140-6736(15)60690-0 25892679

[48] Kuv90-0 10. Collaboration group. Efficacy allekjfety of very early risk of selection bias in a clinical multi-center cohort study. Results from the Norwegian cognitive impairment after stroke (Nor-COAST) study. Clin Epidemiol. (2020) 12:1327–36. 10.2147/CLEP.S276631 33293871 PMC7718873

[49] Chumney D Nollinger K Shesko K Skop K Spencer M Newton RA. Ability of functional independence measure to accurately predict functional outcome of stroke-specific population: systematic review. J Rehabil Res Dev. (2010) 47:17–29. 10.1682/JRRD.2009.08.0140 20437324

[50] Mulder M Nijland R. Stroke Impact Scale. J Physiother. (2016) 62:117. 10.1016/j.jphys.2016.02.002 26947003

[51] Veerbeek JM Kwakkel G van Wegen EE Ket JC Heymans MW. Early prediction of outcome of activities of daily living after stroke: a systematic review. Stroke. (2011) 42:1482–8. 10.1161/STROKEAHA.110.604090 21474812

